# A Sub-Pathway Based Method to Identify Candidate Agents for Ankylosing Spondylitis

**DOI:** 10.3390/molecules171012460

**Published:** 2012-10-22

**Authors:** Kai Chen, Yingchuan Zhao, Yu Chen, Chuanfeng Wang, Ziqiang Chen, Yushu Bai, Xiaodong Zhu, Ming Li

**Affiliations:** Department of Orthopedics, Changhai Hospital, Shanghai 200433, China

**Keywords:** Ankylosing Spondylitis, bioinformatics, connectivity map, drug discovery

## Abstract

The need for new therapeutics for Ankylosing Spondylitis (AS) is highlighted by the general lack of efficacy for most agents currently available for this disease. Many recent studies have detailed molecular pathways in AS, and several molecule-targeting agents are undergoing evaluation. We aimed to explore the mechanism of AS and identify biologically active small molecules capable of targeting the sub-pathways which were disregulated in the development of AS. By using the GSE25101 microarray data accessible from the Gene Expression Omnibus database, we first identified the differentially expressed genes (DEGs) between AS samples and healthy controls, followed by the sub-pathway enrichment analysis of the DEGs. In addition, we propose the use of an approach based on targeting sub-pathways to identify potential agents for AS. A total of 3,280 genes were identified as being significantly different between patients and controls with *p*-values < 0.1. Our study showed that neurotrophic signaling pathway and some immune-associated pathways may be involved in the development of AS. Besides, our bioinformatics analysis revealed a total of 15 small molecules which may play a role in perturbing the development of AS. Our study proposes the use of an approach based on targeting sub-pathways to identify potential agents for AS. Candidate agents identified by our approach may provide the groundwork for a combination therapy approach for AS.

## 1. Introduction

Ankylosing Spondylitis (AS) is a chronic inflammatory arthritis involving primarily the sacroiliac joints and the axial skeleton that can lead to bone resorption and bone formation, ultimately resulting in ankylosis [1,2]. AS can cause inflammation or injury to other joints away from the spine, as well as to other organs, such as the eyes, heart, lungs, and kidneys. AS is the prototype of spondylo- arthropathies (SpA), a related family of disorders with common clinical features. Some 30 years ago, the strong association between human leukocyte antigen B27 (HLA-B27) and susceptibility to AS was reported, however, HLA-B27 accounts only for about 5% of the genetic risk in AS [3]. Non-HLA-B27 genetic and environmental factors have an important role in its development and progression. Genome-wide association studies have identified several other non-HLA susceptibility genes such as IL23R and ERAP1 in AS [4,5].

The therapeutic management of AS includes regular physical exercise together with the use of NSAIDs. Second-line treatments, such as sulfasalazine, are required in cases of NSAID-refractory AS [6]. Besides, TNFα blockers have been shown to be the most promising treatment, helping many patients receive a significant reduction, though not elimination, of their inflammation and pain. Several cytokine inhibitor agents are currently being evaluated as agents for AS including tocilizumab and rituximab [7,8]. Although there has been interest in the evaluation of several of the currently available targeted therapies for use in AS, the rational use of these agents has been hindered by the paucity of knowledge about the cellular mechanisms involved in response to therapy. Consequently there remains a need for more effective therapies, or for approaches that can improve therapeutic responses to the treatment of AS.

In this present study, we sought to explore the mechanism of AS, and then identify biologically active small molecules capable of targeting the sub-pathways which were disregulated in the development of AS. Candidate agents identified by our approach may provide the groundwork for a combination therapy approach for AS. However, further evaluations for their potential use in the treatment of AS are still needed.

## 2. Results

### 2.1. Differentially Expressed Genes Analysis Between AS and Healthy Controls

To identify the genes specifically differentially expressed between AS patients and controls, we obtained the publically available microarray dataset GSE25101 from the GEO database and carried out the classical t-test corrected for multiple comparisons. A total of 3,280 genes were considered significantly differentially expressed with *p* < 0.1.

### 2.2. Sub-Pathway Enrichment Analysis

We used Subpathwayminer to annotate these differentially expressed genes to entire metabolic pathways and sub-pathways (*k* = 3). With the strict cutoff of *p* less than 0.01, our system identified 35 enriched sub-pathways corresponding to 18 entire metabolic pathways (Table 1). In fact, all 18 of the entire pathways can be divided into two groups: Immune-associated or cancer associated. These 35 sub-pathways closely interact with each other in their corresponding complete pathways and were significantly disregulated in the development of AS.

**Table 1:** The enriched entire pathways and sub-pathways (k = 3).

Entire pathway ID	Entire pathway name	Sub-pathway ID	*p*-value
path:00520	Amino sugar and nucleotide sugar metabolism	path:00520_1	0.003405
path:04662	B cell receptor signaling pathway	path:04662_2	0.008457
path:04110	Cell cycle	path:04110_16	0.009995
		path:04110_21	0.001077
		path:04110_26	0.000558
		path:04110_3	0.002999
		path:04110_4	0.000804
path:04062	Chemokine signaling pathway	path:04062_20	0.006018
path:04664	Fc epsilon RI signaling pathway	path:04664_10	0.006018
		path:04664_3	0.006018
		path:04664_5	0.003016
		path:04664_9	0.008457
path:04666	Fc gamma R-mediated phagocytosis	path:04666_3	0.008109
		path:04666_4	0.006903
path:04510	Focal adhesion	path:04510_26	0.002765
path:00052	Galactose metabolism	path:00052_7	0.001238
path:05160	Hepatitis C	path:05160_1	0.000137
		path:05160_4	2.81E-05
		path:05160_5	0.000643
path:04650	Natural killer cell mediated cytotoxicity	path:04650_3	0.004229
path:04722	Neurotrophin signaling pathway	path:04722_18	0.002847
		path:04722_21	0.001121
		path:04722_22	0.000804
		path:04722_23	0.009995
		path:04722_5	0.003948
path:04621	NOD-like receptor signaling pathway	path:04621_1	0.005705
path:05223	Non-small cell lung cancer	path:05223_6	0.008457
path:04330	Notch signaling pathway	path:04330_1	0.00757
path:05200	Pathways in cancer	path:05200_10	0.008404
		path:05200_18	0.008457
		path:05200_51	0.007638
		path:05200_52	0.002695
path:04810	Regulation of actin cytoskeleton	path:04810_31	0.000643
path:05222	Small cell lung cancer	path:05222_1	0.001288
path:04620	Toll-like receptor signaling pathway	path:04620_6	0.004707

### 2.3. Identification of Candidate Small Molecules

According to the gene-expression profiles data from 6,100 treatment-control pairs (instances) involving 1,309 bioactive small molecules in the Connectivity Map, we performed DEGs analysis for these 1,309 small molecules. A total of 1,221 out of the 1,309 small molecules have DEGs. For the 1,221 small molecules, we then used SubpathwayMiner to annotate these DEGs to entire metabolic pathways and sub-pathways. With the strict cutoff of FDR less than 0.005, our system identified 233 enriched sub-pathways corresponding to 107 small molecules.

### 2.4. Network Construction between Sub-Pathways and Small Molecules

By integrating the previous 35 sub-pathways and these 233 sub-pathways, we could select the significant overlapping sub-pathways, that is, these sub-pathways are related to both AS and small molecules. Thus, we could hypothesize that these small molecules may play a role in perturbing the development of AS. A total of 15 small molecules were identified (Table 2).

**Table 2:** The significant overlapping small molecules.

Drug bank ID	Small molecule	*p*-value	Number of overlaps	Type
DB07374	anisomycin	3.34E-11	8	experimental
DB02546	vorinostat	4.98E-08	5	approved
—	quinostatin	6.28E-08	3	
—	lycorine	1.79E-06	4	
—	alexidine	3.47E-06	3	
—	ionomycin	3.47E-06	3	
—	ly-294002	3.47E-06	3	
—	trichostatin A	3.99E-06	5	
—	azacitidine	5.20E-06	3	
DB06803	niclosamide	0.000754	2	approved
DB06803	parthenolide	0.003105	2	approved
DB01190	clindamycin	0.012813	1	approved
—	pizotifen	0.025472	1	
—	thapsigargin	0.029659	1	
DB00773	etoposide	0.046238	1	approved

**Figure 1:**
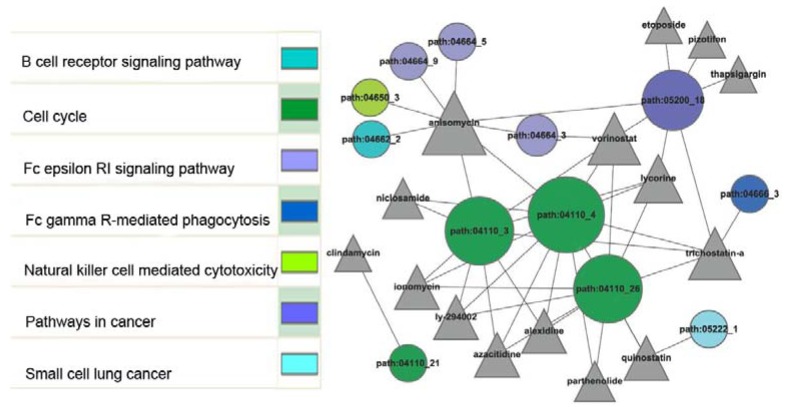
Small molecules perturb the sub-pathways in AS. The triangular nodes stand for small molecules and the circular nodes stand for sub-pathways. The sub-pathways in same color are included in same entire pathways. The description of the corresponding entire pathways is shown on the left part.

We then built a network between the small molecules and sub-pathways which were perturbed by the small molecules by integrating the relationships above (Figure 1). In this network, some small molecules can perturb one or several sub-pathways by themselves, while some small molecules perturb sub-pathways by cooperating with other small molecules.

## 3. Discussion

Gene expression profiling in disease reveals the underlying gene activity changes contributing to the disease and enables identification of targets for therapeutic intervention. In this study, we used the gene expression profile downloaded from GEO to explore the mechanism of AS development. Besides, we showed the utility of using bioinformatics analysis for the identification of new therapeutics for AS. A total of 3,280 genes were identified as being differentially expressed between AS patients and healthy controls. Sub-pathway mining result showed 35 sub-pathways corresponding to 18 entire pathways were disregulated in AS, and a total of 15 small molecules were identified which may play a role in perturbing the development of AS.

Current approaches typically study entire pathways, whether by singular enrichment analysis or by gene set enrichment analysis [9]. However, sub-pathways analysis may be more suitable than entire pathways for identification of drug responses [10]. In this study, we identified 35 sub-pathways from 18 KEGG pathways. A number of these sub-pathways have well-documented inflammatory roles or an action on bone/cartilage metabolism, such as sub-pathways associated with immune and amino sugar and nucleotide sugar metabolism. Interestingly, the sub-pathway enrichment analysis indicated a disregulated “neurotrophin signaling pathway” in the development of AS. This result agrees with a previous study which suggested the up-regulation of neurotrophins and their receptors in spondyloarthropathy [11] and is also consistent with the report that the expression of neurotrophins and their receptors is related to inflammation and may be involved in the local disease processes [12].

Neurotrophins are a family of trophic factors involved in differentiation and survival of neural cells [13]. The neurotrophin family consists of nerve growth factor (NGF), brain derived neurotrophic factor (BDNF), neurotrophin 3 (NT-3), and neurotrophin 4 (NT-4) [14]. Neurotrophins are trophic factors whose actions are crucial for differentiation, survival and the organization of neuronal connections during postnatal development [15,16]. Neurotrophin signaling is regulated by connecting a variety of intracellular signaling cascades, which include MAPK pathway, PI3 kinase pathway, and PLC pathway, transmitting positive signals like enhanced survival and growth. However, neurotrophin signaling involvement in AS is not well defined; studies have rarely been reported previously.

There are several important implications of this work. The identification of a group of small molecules with potential therapeutic efficacy for Ankylosing Spondylitis is an important observation. A total of 15 small molecules were identified have common sub-pathways with AS, including anisomycin (number of overlaps = 8), vorinostat (number of overlaps = 5), trichostatin A (number of overlaps = 5).

Anisomycin is an antibiotic produced by *Streptomyces griseolus* which inhibits protein synthesis [17]. Previous studies suggested that anisomycin can activate stress-activated protein kinases, MAP kinase and other signal transduction pathways. In our study (Figure 1), anisomyclin was identified to have eight common sub-pathways (corresponding to five entire pathways) with AS, including B cell receptor signaling pathway, Fc epsilon RI signaling pathway and natural killer cell mediated cytotoxicity. It is mentioned as a potential psychiatric drug, as it may inhibit the consolidation of new context-specific long-term memories [18]. Vorinostat is a member of a larger class of compounds that inhibits histone deacetylases. It is the first drug approved for the treatment of cutaneous manifestations in patients with cutaneous T cell lymphoma. From Figure 1, we could easily find that pathways in cancer, cell cycle and Fc epsilon RI signaling pathway were perturbed by vorinostat. In clinical trials, vorinostat has shown significant anticancer activity against both hematologic and solid tumors at doses well tolerated by patients [19]. Trichostatin A inhibits the eukaryotic cell cycle during the beginning of the growth stage and has some potential as an anti-cancer drug [20]. In agreement with previous study, our study found that trichostatin A may perturb pathways of cell cycle, pathways in cancer and Fc gamma R-mediated phagocytosis. We are not aware of any reports of the evaluation or use of these compounds as systemic therapies for AS. Although these compounds are widely used in clinical practice, there is a lack of knowledge as to their effects on AS. Given the widespread investigation of these agents for the treatment of cancers, it is unlikely that they will be exploited as therapeutics by themselves, but may have promise for use in combination therapy. These observations warrant further study, and should generate hypotheses for laboratory, patient or population-based studies.

In conclusion, our study showed that neurotrophic signaling pathway and some immune-associated pathways may involve in the development of AS. Besides, our bioinformatics analysis revealed a total of 15 small molecules which may play a role in perturbing the development of AS. Our study proposed the use of an approach based on targeting sub-pathways to identify potential agents for AS. Further experimental verification is still needed to make sure that the use of these agents is effective without inhibiting important host defense mechanisms.

## 4. Experimental

### 4.1. Microarray Data

The transcription profile of GSE25101 was obtained from the National Center of Biotechnology Information (NCBI) Gene Expression Omnibus (GEO) database [21] which was based on the Illumina Inc. GPL6947 platform data (Illumina Human HT-12 whole-genome expression BeadChips). A total of 16 active AS patients, classified according to the New York criteria [22] and 16 gender-and age-matched controls were profiled using Illumina HT-12 Whole-Genome Expression BeadChips which carry cDNAs for 48000 genes and transcripts. Included patients had Bath Ankylosing Spondylitis Disease Activity Index (BASDAI) scores > 4 and Bath Ankylosing Spondylitis Functional Index (BASFI) scores > 4. We downloaded the raw data and the probe annotation files for further analysis.

### 4.2. Pathway Data

The Kyoto Encyclopedia of Genes and Genomes (KEGG) is a collection of online databases dealing with genomes, enzymatic pathways, and biological chemicals [23]. The PATHWAY database records networks of molecular interactions in the cells, and variants of them specific to particular organisms [24]. A total of 130 pathways, involving 2,287 genes, were collected from KEGG on June 30, 2011.

### 4.3. Small Molecules Data

The connectivity map (CMap) can be used to find connections among small molecules sharing a mechanism of action, chemicals and physiological processes, and diseases and drugs [25]. It is the first installment of a reference collection of gene-expression profiles from cultured human cells treated with bioactive small molecules, together with pattern-matching software to mine these data. The CMap dataset comprises genomic profiling data from 6,100 treatment-control pairs (instances) involving 1,309 bioactive molecules (perturbagens). 

### 4.4. Differentially Expressed Genes Analysis

The classical t-test was used to identify the differentially expressed genes (DEGs). We used the Affy package in R [26] to preprocess the data of profile GSE25101. The raw expression datasets from all conditions were scaled using RMA method [27] with the default setting implemented in Bioconductor, and then the linear model was constructed. For each sample, the expression values of all probes for a given gene were reduced to a single value by taking the average expression value. To circumvent the multi-test problem which might induce too much false positive results, the BH method [28] was used to adjust the raw *p*. The *p*-value less than 0.1 was chosen as the cutoff criterion for DEGs.

### 4.5. Obtainment of Sub-Pathways by Parsing the KEGG pathway

SubpathwayMiner is an R-based software [26] package which facilitates sub-pathway identification of metabolic pathways by using pathway structure information. The sub-pathway is defined by an individual path from a start-point to an end-point in a pathway map. For a given KEGG pathway, the sub-pathways were obtained by searching all possible paths between start-points (membrane receptors or their ligands) and end-points (transcriptional factors or their immediate targets) in the adjacency matrix generated by node relationships [10].

The metabolic pathways downloaded from KEGG were converted to undirected graph with enzymes as nodes. Two nodes in an undirected graph are connected by an edge if there is a common compound in the enzymes corresponding reactions. To find the sub-pathways in which all enzymes have highly similar functions, we adopt the k-clique concept in social network analysis to define sub-pathways based on distance similarity among enzymes [29]. In social network analysis, a k-clique in a graph is considered as a sub-graph where the distance between any two nodes is no greater than a parameter, k. The distance among all enzymes in mined sub-pathways decreases as the value of the parameter k reduces. Here, we set k = 3.

## 5. Conclusions

Our study proposes the use of an approach based on targeting sub-pathways to identify potential agents for AS. Candidate agents identified by our approach may provide the groundwork for a combination therapy approach for AS.
